# Epigenetics, Modifiers, and Molecular Noise: Rethinking Pathophysiology in Dilated and Hypertrophic Cardiomyopathies

**DOI:** 10.3390/ijms27073159

**Published:** 2026-03-31

**Authors:** Miruna Mihaela Micheu, Eugeniu Catlabuga, Alexei Leahu, Dumitru Ciorbă, Viorel Munteanu

**Affiliations:** 1 Department of Cardiology, Clinical Emergency Hospital of Bucharest, 8, Calea Floreasca, 014461 Bucharest, Romania; 2 Department of Computers, Informatics and Microelectronics, Technical University of Moldova, 2045 Chișinău, Moldova; eugeniu.catlabuga@doctorat.utm.md (E.C.); alexei.leahu@ati.utm.md (A.L.); dumitru.ciorba@fcim.utm.md (D.C.); viorel.munteanu@lt.utm.md (V.M.)

**Keywords:** hypertrophic cardiomyopathy, dilated cardiomyopathy, epigenetics, modifier, molecular noise

## Abstract

Cardiomyopathies comprise a heterogeneous group of myocardial disorders characterized by intrinsic structural and functional abnormalities that are not explained by secondary cardiovascular or systemic conditions. Although genetically determined cardiomyopathies have traditionally been interpreted within a Mendelian framework, this paradigm does not fully account for the marked variability in penetrance, expressivity, and clinical outcomes observed in affected individuals. Increasing evidence indicates that disease manifestation arises from a complex interplay between rare pathogenic variants, common genetic variation, epigenetic regulation, environmental factors, and stochastic molecular processes. This review focuses on hypertrophic and dilated cardiomyopathies, the most prevalent and extensively studied forms, and critically examines how epigenetic mechanisms, genetic modifiers, and molecular noise challenge classical pathophysiology concepts. We discuss how these factors contribute to phenotypic heterogeneity and influence disease severity, progression, and therapeutic response. Recognition of this multilayered genetic architecture has important clinical implications, supporting more refined risk stratification, improved genetic counseling, and the development of personalized and potentially variant-agnostic therapeutic strategies.

## 1. Introduction

Cardiomyopathies (CMPs) comprise a heterogeneous group of myocardial disorders defined by intrinsic structural and functional abnormalities of the cardiac muscle, that arise in the absence of alternative systemic or cardiovascular conditions capable of producing the same phenotype, including coronary artery disease, hypertension, valvular pathology, or congenital heart defects [1]. Clinically, CMPs are classified into five major disease phenotypes: hypertrophic cardiomyopathy (HCM, OMIM #192600), dilated cardiomyopathy (DCM, OMIM #115200), arrhythmogenic right ventricular cardiomyopathy (ARVC, OMIM #107970), restrictive cardiomyopathy (RCM, OMIM #115210), and the more recently delineated non-dilated left ventricular cardiomyopathy (NDLVC) [1].

**Figure 1:**
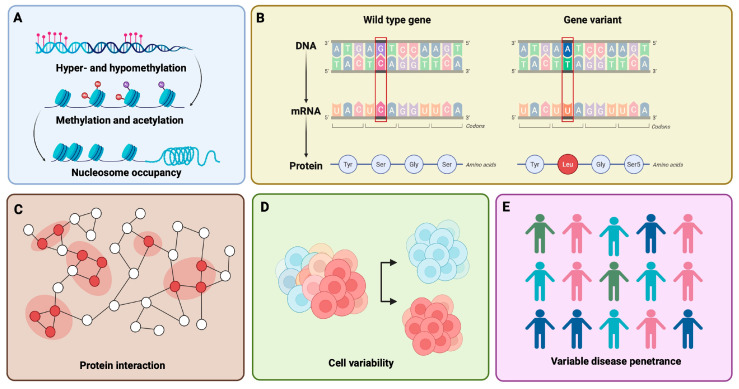
Determinants of molecular variability and disease penetrance in cardiomyopathies. (**A**) Epigenetic mechanisms (DNA methylation, histone marks, chromatin structure) regulate cardiomyopathy gene expression without altering DNA sequence. (**B**) Pathogenic mutations and genetic variation in core cardiac genes alter protein structure and function. (**C**) Genetic modifiers and stochastic gene expression propagate variability through molecular interaction networks. (**D**) Regulatory variability generates cell-to-cell differences in cardiomyocyte states and function. (**E**) The combined effects of mutations, modifiers, epigenetics, and noise result in variable penetrance and clinical heterogeneity. Created in BioRender. Munteanu, V. (2026) https://BioRender.com/aqcm15n.

Historically, CMPs with a genetic aetiology have been conceptualized within the framework of classical Mendelian inheritance, most often as autosomal dominant disorders, but also including autosomal recessive, X-linked, and mitochondrial inheritance patterns. This monogenic paradigm enabled the discovery of core disease genes, notably sarcomeric variants in HCM and desmosomal defects in ARVC, and established the foundational links between genotype, myocardial architecture, and clinical phenotype [2]. Nevertheless, the Mendelian model alone appears insufficient to explain the full clinical and genetic complexity observed in many patients. Variable penetrance, intrafamilial phenotypic heterogeneity, and incomplete genotype–phenotype correlations are frequently encountered. These features challenge the monogenic paradigm and instead point to a more complex genetic architecture involving polygenic influences, modifier genes and variants, epigenetic regulation, and stochastic variation (Figure 1) [3,4].

Genetically determined CMPs arise from pathogenic variation in genes encoding key components of the cardiac sarcomere, cytoskeleton, nuclear envelope and associated regulatory pathway [5,6]. Among these conditions, HCM and DCM represent the most prevalent and best-characterized conditions in clinical genetics. Large-scale gene–disease curation efforts and international consensus frameworks have established a set of genes with robust evidence supporting their causal role in CMPs, with genes classified as having definitive, strong or moderate gene–disease validity for HCM [7] and DCM [8] (Table 1).

Most genes associated with HCM encode sarcomeric proteins responsible for force generation and contractile regulation, whereas DCM genes more frequently involve proteins involved in cytoskeletal organization, nuclear envelope integrity, ion transport or transcriptional control of cardiomyocyte structure. These distinctions reflect the underlying biological processes that govern myocardial contraction, cellular architecture and mechanical stress responses in cardiac tissue.

**Figure 2:**
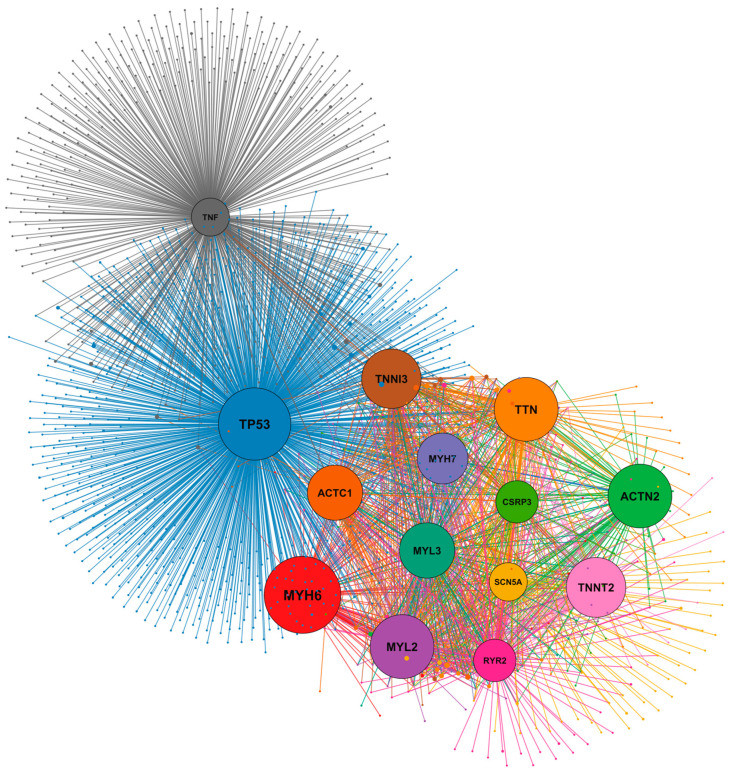
Protein–protein interaction network showing the connectivity of cardiomyopathy-associated genes analyzed in this paper and their interacting partners retrieved from curated interaction databases using the STRING database (version 12.0) [9] and visualized in Cytoscape (version 3.10.4) [10]; central nodes represent core disease genes, peripheral nodes correspond to interacting proteins, and node size reflects network degree and connectivity within the interaction landscape.

Network-based analyses indicate that several canonical cardiomyopathy genes act as highly connected hubs within protein–protein interaction networks that integrate sarcomeric architecture with calcium handling, electrophysiological signaling and cellular stress-response pathways (Figure 2). Through their extensive interactions with numerous secondary proteins, these hub genes contribute to the regulation of contractile mechanics, excitation–contraction coupling and cardiomyocyte adaptation to physiological and pathological stress, underscoring the systems-level organization of the molecular networks underlying cardiomyopathy pathogenesis.

**Figure 3:**
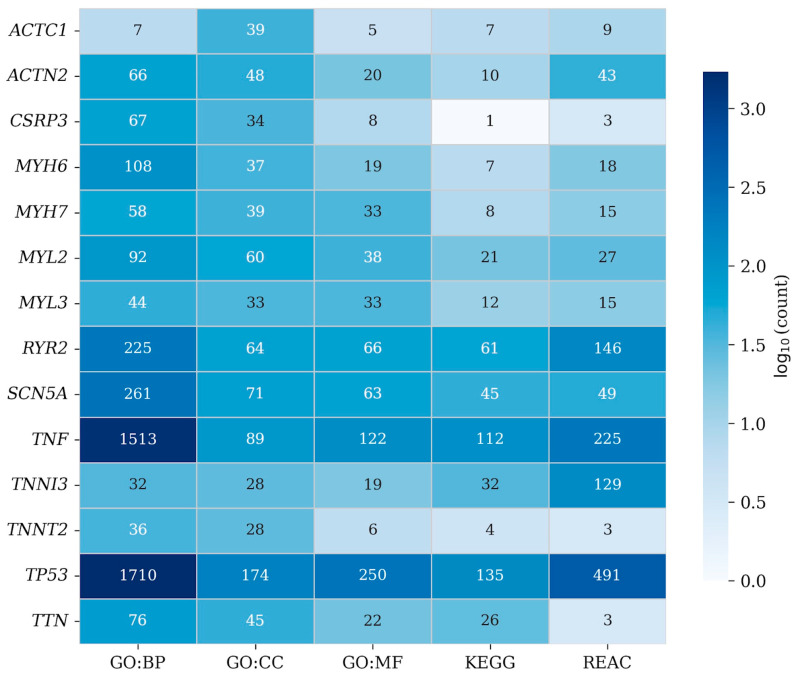
Heatmap summarizing the functional annotation profiles of cardiomyopathy-associated genes analyzed in this study across Gene Ontology biological processes (GO:BP), cellular components (GO:CC), molecular functions (GO:MF), KEGG pathways and Reactome pathways, illustrating the breadth and diversity of molecular functions and pathways in which these cardiomyopathy-related genes participate.

Functional enrichment analyses further highlight the broad biological landscape associated with cardiomyopathy genes (Figure 3). Gene ontology annotations demonstrate strong enrichment for biological processes related to cardiac muscle contraction, cytoskeletal organization and sarcomere assembly, whereas pathway analyses from KEGG and Reactome identify signaling cascades involved in calcium homeostasis, cardiomyocyte remodeling and inflammatory responses. Notably, genes such as *TP53* and *TNF* exhibit extensive functional connectivity across diverse biological pathways, suggesting that cellular stress responses and inflammatory signaling may contribute to the molecular context that modulates cardiomyopathy phenotypes.

In this review, we examine the emerging roles of epigenetic regulation, genetic modifier variants and stochastic molecular processes in shaping the phenotypic expression of cardiomyopathies. We discuss how these mechanisms influence disease penetrance, severity and phenotypic heterogeneity, thereby extending the classical Mendelian framework and providing a more comprehensive view of CMP pathophysiology beyond strictly monogenic models.

## 2. Epigenetic Regulation in Cardiomyopathies

Epigenetic mechanisms are heritable yet reversible modifications to chromatin and RNA that regulate gene expression without altering DNA sequence, and they have emerged as key mediators of phenotypic variability in genetically determined CMPs. Traditional gene-centric models explain disease by pathogenic variants in coding sequences, yet growing evidence indicates that DNA methylation, histone post-translational modifications, and higher-order chromatin architecture modulate the penetrance, expressivity and progression of these cardiomyopathies [11,12,13,14,15]. These epigenetic alterations affect regulatory regions and structural genes involved in myocardial contractility, electrical signaling and cardiac remodeling. Numerous loci and regulatory pathways implicated in these processes have been identified in dilated and hypertrophic cardiomyopathies, including genes associated with sarcomeric function, ion-channel regulation and chromatin-remodeling complexes (Table 2).

### 2.1. DNA Methylation and Cardiomyopathy-Specific Signatures

Epigenome-wide and targeted methylation analyses of left ventricular (LV) tissue from patients with inherited CMPs revealed altered DNA methylation patterns. Differentially methylated regions span promoters, introns and distal enhancers of genes governing sarcomeric architecture, ion homeostasis, and extracellular matrix organization, often correlating with altered transcript levels.

#### 2.1.1. DNA Methylation in Hypertrophic Cardiomyopathy

Integrated DNA methylation (DNAme) and transcriptome profiling of HCM myocardium has revealed widespread epigenetic alterations associated with impaired myocardial function [16]. Aberrant hypermethylation and hypomethylation were found to affect gene expression and disrupt key cardiac cellular processes involved in electrophysiology, immune signalling and cardiomyocyte (CM) structural integrity. The chromosomal distribution and functional enrichment of these methylation sites in HCM tissues differed markedly from those in normal myocardium, pointing to disease-specific epigenetic remodeling. Gene Ontology (GO) analyses showed that loci exhibiting changes in DNAme methylation and expression were predominantly involved in immune regulation and muscle system processes. Pathway analysis further revealed that calcium signalling was the only KEGG pathway consistently enriched in both methylation-associated and differentially expressed gene sets. Protein–protein interaction analysis of genes showing both DNA methylation and transcriptional changes revealed two major functional clusters, one related to tissue development and immune regulation (*ESR1*, *STC2*, *TGFA*, *TGFB2*, and *VEGFC*) and another centered on cardiac electrophysiology, with *CACNA1A* as a central node. Integrating DNA methylation with transcriptomic profiles provides valuable insights into the regulatory networks perturbed in HCM pathogenesis and may facilitate the identification of novel therapeutic targets. Consistent with this framework, distinct differentially methylated regions (DMRs) have been observed in HCM myocardium even in absence of major shifts in global methylation levels, indicating localized epigenetic remodeling with functional consequences [38]. Specifically, 1453 hypermethylated DMRs were found in HCM myocardium, mapping on genes involved in actin filament organization, blood circulation, and cardiac contraction, whereas 3600 hypomethylated DMRs were associated with pathways related to synapse organization, cell adhesion, and intercellular signaling. These DMRs were predominantly enriched in exons, promoters, and enhancers, and despite the overall similarity in methylation patterns across these regions, targeted analysis revealed specific alterations distinguishing HCM patients from controls. For instance, *TPM1*, the gene coding alpha-troposin, a thin-filament regulatory protein implicated in HCM and congenital heart malformations, shows promoter hypermethylation in affected myocardium [17]. Functional evidence supporting this comes from the E192K *TPM1* mutation, where loss of cross-bridge inhibitory control produces pathological hypertrophy through increased thin-filament activation [43]. Consistent with these findings, HCM tissue displays increased gene-body methylation in other sarcomeric regulators such as *TCAP* and *ACTA1*, both of which encode sarcomeric proteins linked to CMPs [18,19]. Interestingly, despite the transcriptomic evidence of fetal gene reprogramming in HCM, characterized by reduced expression of sarcomeric and metabolic genes and increased expression of extracellular matrix components, the global levels of DNA methylation in adult controls, HCM myocardium, and fetal hearts were comparable, suggesting that the myocardial DNA methylome remains relatively stable compared to the more dynamic transcriptome and chromatin landscape, reflecting its fundamental role in maintaining genomic integrity [44].

Primary and secondary forms of cardiac hypertrophy can be distinguished by their distinct transcriptional and epigenetic architectures [45]. By using aortic stenosis (AS) samples as a control, the authors minimized confounding adaptive hypertrophic responses and thereby delineated HCM-specific transcriptional and epigenetic signatures. Distinct DNA methylation profiles clearly differentiated HCM from AS, supporting a contributory role of epigenetic regulation in the development of primary hypertrophy [45]. Integrative methylation-expression analysis identified eight genes whose expression correlated with neighboring hypomethylated CpG sites within their gene bodies, indicating locus-specific regulatory effects [20]. Four of these genes (*AUTS2*, *BRSK2*, *PRRT1*, and *SLC17A7*) are implicated in neurodevelopment and synaptic signaling pathways, suggesting that perturbations in neuro-cardiac communication may form part of the HCM pathophysiological signature. The remaining genes (*GIGYF1*, *CBFA2T3*, *HIVEP3*, and *ANKRD33B*) are involved in receptor signaling, transcriptional regulation, or remain functionally uncharacterized. In addition to these locus-specific effects, further analyses of the HCM methylome identify four genomic regions showing reduced DNA methylation in diseased myocardium, encompassing intragenic and promoter-proximal loci linked to *PAX8*, *PSORS1C3*, *GATA4*, and *PIWIL2* [20]. Among these, *PAX8* and *GATA4* showed altered expression, suggesting that hypomethylation at these sites may contribute to transcriptional dysregulation of key developmental and cardiac transcription factors.

Collectively, these findings highlight the contribution of DNA methylation to HCM pathogenesis and identify novel gene candidates potentially driving disease-specific myocardial remodeling.

#### 2.1.2. DNA Methylation in Dilated Cardiomyopathy

DCM constitutes one of the most prevalent forms of CMP; consequently, it has been the focus of numerous studies examining epigenetic remodeling in this context [21,22,23,46]. Notably, cytosine methylation profiles in DCM differ markedly from those observed in other forms of heart failure, delineating disease-specific epigenetic signatures with potential diagnostic and therapeutic significance.

Haas and colleagues conducted the first genome-wide analysis of cardiac DNA methylation in LV tissue from patients with non-ischaemic, idiopathic DCM [21]. Their study identified several candidate genes with altered methylation profiles, findings subsequently validated in an independent cohort of DCM patients and controls. Although overall methylation patterns were largely preserved between groups, specific CpG islands were found to be significantly hypo- or hypermethylated in DCM compared with controls. Notably, distinct methylation signatures were observed in genes not previously implicated in DCM pathophysiology, including *LY75* (Lymphocyte antigen 75), *ERBB3* (Tyrosine kinase-type cell surface receptor HER3), *HOXB13* (Homeobox B13), and *ADORA2A* (Adenosine receptor A2A). Complementary gene expression analyses and functional assays in zebrafish demonstrated that appropriate expression of *LY75* and *ADORA2A* is essential for normal cardiac function. Collectively, these findings indicate that aberrant DNA methylation modulates gene expression and may contribute to the phenotypic manifestations of idiopathic DCM.

Four years later, the same German research group performed a high-resolution, epigenome-wide analysis of DNA methylation in cardiac and peripheral blood samples from a large cohort of deeply phenotyped patients with DCM and systolic heart failure. This multi-omic approach, integrating mRNA expression and whole-genome sequencing data, enabled the identification of disease-specific cardiac gene patterning and the discovery of a novel class of biomarkers [22]. In the screening cohort, the investigators identified 59 CpG loci exhibiting differential methylation in the myocardium of patients with DCM (*n* = 41) compared to controls (*n* = 31). Of these, 30 loci were hypomethylated and 29 hypermethylated, with three—cg16318181, cg01977762, and cg23296652—reaching epigenome-wide statistical significance. Moreover, comparative analyses of methylation profiles from myocardial tissue and peripheral blood in both the discovery and validation DCM cohorts demonstrated significant overlap for three CpG sites: two hypomethylated (cg24884140, located in *B9D1*; and cg12115081, within *DCLK2*) and one hypermethylated (cg25943276, associated with *NTM*). The combined use of these three markers demonstrated excellent diagnostic performance for DCM, achieving an area under the curve (AUC) of 0.915 in the screening cohort and 0.869 in the replication cohort—exceeding that of NT-proBNP (AUC = 0.85), a conventional biomarker of heart failure. An important aspect of the study consists in the identification of six genomic regions that were found to be significantly differentially methylated in DCM. These regions encompass a considerable number of genes involved in cardiac development, heart function, and cardiomyopathies, with 9 out of 72 dysmethylated genes showing established associations with such processes. One particularly notable finding concerns the locus 12q24.21, which includes *MED13L*, *TBX3*, *TBX5*, *TBX5-AS1*, *RBM19*, and microRNA 620—all affected by significant methylation changes. Among these, *TBX5*, encoding a T-box transcription factor, is essential for cardiogenesis [47], and mutations in this gene have been identified in both familial and sporadic DCM [48]. Similarly, *MED13L* gene, a component of the Mediator complex, plays a critical role in cardiovascular development [49], with disruptions leading to congenital cardiac abnormalities [50].

Additionally, the study identified *MYL2*, located about 3 Mb upstream at 12q24.11, as a dysmethylated gene in DCM. *MYL2* encodes the ventricular regulatory myosin light chain, a key sarcomeric protein in early cardiac development and one of the earliest markers of ventricular specification. Mutations in this gene have been linked to dilated and hypertrophic CMP, indicating that its altered methylation may contribute to ventricular dysfunction [51].

In a recent study, Tan and colleagues conducted the most extensive epigenome-wide association study (EWAS) to date focusing on DCM using cardiac tissue samples [23]. The discovery cohort included 159 DCM cases and 170 controls, representing a substantial expansion in both sample size and CpG site coverage compared to previous EWAS investigations on DCM. During the discovery phase, the authors identified 36,925 CpG sites significantly associated with DCM (FDR *p* < 0.05), which were subsequently evaluated for replication in an independent DCM cohort, thereby strengthening the robustness of their findings. In addition to confirming associations previously reported by earlier EWAS studies of DCM, the authors identified a putative causal relationship between cg08140459 and DCM. Their analysis demonstrated that cg08140459 was strongly associated with the expression of *LTBP2*, a recently recognized prognostic biomarker for DCM, across independent cohorts [52], thereby reinforcing the biological plausibility of this link. Furthermore, to elucidate the molecular mechanisms underlying DCM pathogenesis, the investigators performed a summary-based Mendelian randomization (SMR) analysis focused on gene expression. This approach revealed the potential involvement of two additional sentinel CpG sites—cg11793257 (associated with *ABHD12*) and cg01651169 (associated with *ATP5MF*)—both genes implicated in pathways relevant to cardiac remodeling and energy metabolism. These findings highlight novel epigenetic-gene expression interactions that may contribute to DCM progression and merit further validation in dedicated cardiac studies.

Another study explored the epigenetic contribution to DCM by profiling DNA methylation (5mC) and hydroxymethylation (5hmC) in a *MYBPC3* mutant mouse model versus wild-type controls [53]. They identified 957 genes with altered 5mC and 2022 genes with altered 5hmC in DCM hearts. Enrichment analyses (GO, KEGG) implicated processes and pathways relevant to DCM—including inflammation, fibrosis, cell death, cardiac remodeling, CM growth/differentiation, and sarcomere organization—while hierarchical clustering of affected genes robustly separated DCM from WT samples. These results support a central role for genome-wide 5mC and 5hmC alterations in DCM pathogenesis.

DNA methylation influences transcript splicing and may promote pathological exon-skipping, a key mechanism in DCM pathogenesis. Intronic methylation within the *TTN-AS1* gene, which encodes the long noncoding RNA Titin antisense 1, shows a strong association with inclusion of flanking exons [24]. These differentially methylated regions overlap with the titin A-band locus, commonly affected by DCM-related mutations [25,26]. Aberrant *TTN-AS1* splicing may therefore recapitulate defects observed in *TTN* truncating variants, suggesting that epigenetic modulation of this locus could offer a novel therapeutic target [54,55].

Tappu et al. (2022) identified a novel gene network connecting sarcomeric and membrane-associated components via CpG methylation-mediated regulation [56]. Specifically, the key sarcomeric genes *NEB* and *TPM3*, together with *ERC2*, a Rab-interacting molecule (RIM) family gene not previously implicated in CMP but known to modulate voltage-gated Ca^2+^ channels [57], showed significant associations with CpG methylation sites within the *LRRC14B* locus. This finding implicates *LRRC14B*, a leucine-rich repeat-containing gene, as a potential epigenetic hub in DCM. Comparable but weaker correlations were also observed at the *LRRC10* locus, another member of the same gene family with a well-documented role in DCM pathogenesis [58,59]. Additional interactions between sarcomeric and ion transporter/membrane protein genes, including *ATP11A*, *SLC12A7*, and *TSPAN9*, suggest that epigenetic co-regulation may integrate contractile, metabolic, and electrophysiological remodeling in DCM.

Particular attention has been directed toward forms of DCM associated with mutations in the *LMNA* gene. According to ClinGen, *LMNA* has definitive evidence for causing DCM [60], and among all genetic forms, *LMNA*-related DCM accounts for up to 10% of cases, representing the second most prevalent genetic cause after *TTN*-associated disease. This disorder typically exhibits high penetrance and early onset, and is frequently associated with conduction system disturbances and malignant arrhythmias, often progressing to advanced heart failure [61,62,63,64,65,66]. These features underscore its major contribution to the genetic spectrum of DCM and its distinct clinical and prognostic implications within this heterogeneous disorder.

The *LMNA* gene encodes lamin A/C, a structural component of the nuclear lamina that play a pivotal role in chromatin organization and transcriptional regulation through lamin-associated domains (LADs) [67,68]. In human CMs, *LMNA* interacts with approximately 20% of the genome, modulating the expression of thousands of genes [69]. LADs generally repress transcription via CpG methylation and recruitment of inhibitory epigenetic marks. Thus, *LMNA* mutations may alter LAD positioning, leading to transcriptional silencing of genes crucial for cardiac electrical stability, such as *SCN5A* [27]. Cheedipudi et al. [28] further demonstrated that LAD reorganization in LMNA-associated DCM is associated with changes in CpG methylation and extensive dysregulation of coding and noncoding genes involved in cell death/survival, cell cycle regulation, and metabolic pathways, highlighting the epigenetic consequences of *LMNA* dysfunction. Specifically, the authors identified *TP53* as a major activated signaling pathway in cardiomyocytes from their patients. Interestingly, examination of publicly available RNA-seq datasets from patients with non-LMNA forms of heart failure, including ischemic cardiomyopathy, did not reveal *TP53* activation, indicating that induction of this pathway may be a specific feature of laminopathy-related DCM rather than a general response to cardiac stress.

Taken together, these findings highlight the potential role of DNA methylation in the pathogenesis of genetic CMPs, suggesting that methylation-mediated regulation of cardiac developmental and structural genes contributes substantially to disease manifestation and progression.

### 2.2. Histone Modifications and Chromatin Architecture Alteration

Emerging evidence indicates that histone modifications and chromatin architecture remodeling are critical epigenetic mechanisms driving the development and progression of CMPs through dysregulation of cardiac gene expression [15]. Histone modifications are covalent post-translational changes that regulate chromatin structure and gene expression, including acetylation, methylation, phosphorylation, ubiquitylation, and sumoylation [70]. Histone acetylation of lysine residues promotes chromatin relaxation and transcriptional activation through the coordinated activity of histone acetyltransferases (HATs) and histone deacetylases (HDACs) [71]. Histone methylation modulates gene expression depending on the specific modified amino acid residue and the degree of methylation. Transcriptional activation is typically associated with di- or tri-methylation of histone H3 at lysines 4, 36, and 79 (H3K4, H3K36, and H3K79, respectively), as well as monomethylation of H3K9 and H4K20. In contrast, transcriptional repression correlates with trimethylation of H3K9, H3K27, and H4K20, or with demethylation of H3K9. These repressive marks contribute to gene silencing by promoting the formation of facultative or constitutive heterochromatin. Histone methylation and demethylation are enzymatically regulated by histone methyltransferases (HMTs) and histone demethylases (HDMs), respectively [72]. The dynamic organization of chromatin—ranging from euchromatin, which permits transcriptional activation, to heterochromatin, which enforces gene silencing—enables precise spatiotemporal regulation of gene expression. Disruption of chromatin organization can therefore lead to aberrant gene expression and contribute to disease pathogenesis [73,74].

#### 2.2.1. Histone Modifications and Chromatin Architecture Alteration in Hypertrophic Cardiomyopathy

Ackerman’s group demonstrated extensive dysregulation of histone architecture in myectomy tissue from patients with obstructive HCM (HOCM), with enrichment of histone modifications at cardiac hypertrophy-associated loci, including RAS–MAPK signaling regions, characterized by both activating marks (H3K27ac and H3K9ac) and repressive marks (H3K27me) [29]. These findings build upon the group’s previous work, which characterized the ventricular proteome in HOCM cardiac tissues and demonstrated that, independent of genotype, there is a pervasive upregulation and activation of hypertrophic signaling pathways, predominantly involving the RAS–MAPK cascade [30]. Notably, this proteomic activation was accompanied by a counterregulatory transcriptional downregulation of the same pathways, indicating a complex, multilayered regulatory landscape. The authors proposed that this transcriptional suppression may represent a protective feedback mechanism aimed at restraining excessive cardiac hypertrophy and limiting disease progression. The present data, demonstrating the coexistence of activating and repressive histone modifications at these loci, provide a plausible epigenetic basis for this apparent discordance between transcriptional repression and proteomic activation.

Integrated multi-omics analyses comparing HCM patients, healthy adults, and fetal hearts revealed that HCM myocardium partially reverts to a fetal-like chromatin accessibility pattern at specific genomic loci [38]. Several transcription factors (TFs) displayed fetal-type motif enrichment, among which *SP1* and *EGR1* emerged as key regulators. Both TFs were upregulated and associated with increased chromatin accessibility in HCM samples. Pharmacological inhibition of *SP1* (plicamycin) or *EGR1* (ML264) significantly diminished cardiac hypertrophy in HCM mouse models, suggesting that targeting these fetal-pattern TFs may mitigate pathological remodeling by reversing fetal transcriptional reprogramming.

*BRG1*, a core subunit of the SWI/SNF chromatin-remodeling complex, plays a crucial role in cardiac development by repressing α-MHC (*MYH6*) and activating β-MHC (*MYH7*) expression during fetal cardiomyocyte differentiation [31]. Normally silenced in the adult heart, *BRG1* is reactivated in HCM, where it promotes a reversion to embryonic-like gene expression patterns through α/β-MHC isoform switching. Elevated *BRG1* levels have been observed in both patient samples and experimental models, suggesting aberrant chromatin remodeling contributes to HCM pathogenesis [31,32]. Indeed, previous studies investigating histone modifications and chromatin dynamics in pathologically stressed murine hearts have shown that *BRG1* activation promotes the recruitment of the histone methyltransferase G9a and the DNA methyltransferase DNMT3, resulting in the enrichment of repressive marks (H3K9me2 and CpG methylation, respectively) on the *Myh6* promoter. These epigenetic modifications lead to *MYH6* silencing and a subsequent decline in cardiac contractile function [75].

#### 2.2.2. Histone Modifications and Chromatin Architecture Alteration in Dilated Cardiomyopathy

Montgomery’s study provided compelling evidence for the overlapping yet distinct roles of HDAC1 and HDAC2 in cardiac development and function [34]. The findings emphasise a critical dosage-dependent requirement for these deacetylases, as the individual, cardiac-specific loss of either enzyme is well tolerated, whereas their combined deletion led to structural and functional abnormalities resembling a DCM phenotype, characterized by severe ventricular dilation and arrhythmias. The concomitant loss of HDAC1 and HDAC2 induced a highly specific transcriptional response, including the selective up-regulation of genes involved in calcium influx and homeostasis (e.g., *CACNA1H*, *CACNA2D2*), as well as the aberrant activation of skeletal muscle contractile protein genes in the myocardium (*ACTA1*, *MLC1F*, *TNNT3*, *TNNI1*, *TNNI2*). This transcriptional reprogramming perturbed calcium handling and cardiac contractility, contributing to the observed pathological phenotype.

Ito and colleagues reported significant alterations in histone modification patterns in patients with end-stage non-ischemic DCM [76]. Specifically, a marked decrease in H3K4me3, H3K9me2, and H3K9me3 levels was observed compared with normal LV tissue, suggesting a link between global histone modification changes and the pathogenesis of DCM. Notably, the expression of natriuretic peptides (both ANP and BNP) was inversely correlated with the levels of H3K9me2 and H3K9me3. Furthermore, implantation of a LV assist device (LVAD) appeared to reverse these epigenetic alterations, as evidenced by the upregulation of two methyltransferases (H3K9 methyltransferase and SUV39H1) and the downregulation of two demethylases (H3K9 demethylase and jumonji domains- JMJDs).

DOT1L, which catalyses the methylation of histone H3 at lysine 79, is another histone methyltransferase that has been investigated in relation to the pathogenesis of DCM [35]. Reduced DOT1L expression has been observed in DCM hearts, while cardiac-specific Dot1L knockout mice develop a DCM phenotype characterized by chamber dilation, increased cardiomyocyte death, systolic dysfunction, conduction abnormalities, and elevated mortality. Further analyses have shown that DOT1L regulates the transcription of the Dystrophin (Dmd) gene, thereby maintaining the stability of the dystrophin–glycoprotein complex, which is crucial for cardiomyocyte structural integrity [36]. Notably, expression of a miniaturized Dmd construct (miniDmd) markedly alleviated the DCM phenotype, supporting the notion that Dmd functions as a key downstream effector of DOT1L in CMs [35].

Geng et al. demonstrated that expression profiles of chromatin structural genes robustly stratified myocardial tissue according to disease status [37]. DCM CMs displayed reduced contractile gene expression and increased transcription of cardiomyopathy-associated markers. Among chromatin regulators, HMGN3 was consistently downregulated in DCM CMs and in experimental heart failure models. HMGN3, a member of the High Mobility Group Nucleosome-binding (HMGN) family, functions as a non-histone chromatin architectural protein that modulates nucleosome dynamics and counteracts histone H1 binding, thereby reducing chromatin compaction and enhancing chromatin accessibility [77]. Functional analyses in AC16 human cardiomyocytes demonstrated that HMGN3 loss triggered extensive transcriptional reprogramming, affecting over 2800 genes, and altered H3K27ac distribution, consistent with chromatin reorganization. Loss of HMGN3 exerted bidirectional transcriptional effects, simultaneously inducing and repressing distinct gene networks, including compensatory upregulation of chromatin-remodelling factors (e.g., *HMGA1/2*, *ZMYND8*, *DPF3*, *H2BC7*, *H3C12*, *H4C16*). These changes were accompanied by activation of inflammatory *(IL1A*, *IL1B*, *CXCL8*), hypoxic (*VEGFA*, *BNIP3*, *CA9*, *SLC2A1*), and apoptotic (*BNIP3*, *TP53I3*, *GLIPR1*) pathways, alongside suppression of survival (*FAS*, *BBC3*, *BTG2*), cardiac structural (*DES*, *ACTA2*, *LMOD1*, *TMOD1*, *OBSCN*, *MYOM1*, *PKP2*), and contractile genes (*KCNJ11*, *KCNJ14*, *CACNA2D2*) [37]. Collectively, these findings position HMGN3 as a central modulator of chromatin architecture, linking chromatin remodeling to maladaptive transcriptional reprogramming and cardiac dysfunction in DCM.

Emerging evidence demonstrates that *LMNA*-mutation cardiomyopathy involves profound alterations in chromatin architecture and histone modification profiles. For example, in human cardiomyocytes with *LMNA*-associated DCM, LADs were extensively redistributed, with approximately 520 gained and 149 lost relative to controls. This LAD reorganization coincided with altered CpG methylation and widespread dysregulation of genes implicated in metabolism, cell cycle regulation, and cell death [28]. Moreover, alterations in chromatin architecture were accompanied by dysregulation of multiple ion channel genes, including *KCNA5*, *SCN5A*, *CACNA1A/C/D*, *HCN4*, *SCN3B*, and *SCN4B*, which may contribute to the rhythm and conduction disturbances observed in patients with *LMNA* mutations [27,39,40,41,42].

Human induced pluripotent stem cell-derived cardiomyocytes (hiPSC-CMs) carrying the *LMNA* K117fs frameshift mutation, which causes lamin A/C haploinsufficiency, exhibited increased open chromatin in LADs, resulting in aberrant activation of the *PDGF* signaling pathway [40]. Hyperactivation of this pathway contributed to arrhythmic phenotypes, while pharmacological inhibition of PDGF receptors (with crenolanib or sunitinib) or siRNA-mediated silencing of PDGFRB restored normal electrophysiological properties. These findings suggest a therapeutic potential for *PDGF* pathway blockade in *LMNA*-related cardiomyopathy. Interestingly, the study also revealed transcriptional activation of genes in non-LAD regions, indicating that *LMNA* haploinsufficiency drives a broader, maladaptive epigenetic reorganization beyond lamina-associated chromatin [40].

Zhu et al. investigated the molecular mechanisms underlying atrial arrhythmogenesis associated with *LMNA* mutations using a heterozygous knock-in mouse model carrying the K418Sfs frameshift mutation [42]. These mice developed progressive, spontaneous atrial arrhythmias accompanied by increased atrial fibrosis, while ventricular structure and function remained unaffected—closely recapitulating the phenotype observed in diseased subjects harboring the aforementioned variant. Transcriptomic and chromatin analyses revealed pronounced downregulation of the atrial-specific potassium channel gene *KCNA5*, correlated with increased lamin A/C binding and reduced interaction with the activating histone mark H3K4me3 in mutant atria [42].

H3K4me3 represents the principal substrate of the histone lysine demethylase 5 (KDM5) family, which has been shown to be activated in cardiomyocytes (CMs) in a mouse model of lamin-associated CMP [78]. Among these enzymes, KDM5A has been particularly implicated in the pathogenesis of *LMNA*-related DCM. Upregulation of *KDM5A* expression correlated with repression of its downstream targets, including genes involved in mitochondrial biogenesis and function. Conversely, genetic deletion of KDM5A prolonged survival, alleviated cardiac dysfunction, and promoted the expression of genes associated with oxidative phosphorylation (OXPHOS) and fatty acid oxidation (FAO) [79]. Consistent with these findings, recent studies using mouse iPSC-derived CMs have revealed enrichment of H3K4me3 peaks at promoter regions of genes encoding FAO, OXPHOS, and sarcomeric proteins. Inhibition of KDM5 activity boosted the maturation of iPSC-CMs by epigenetically upregulating OXPHOS-, FAO-, and sarcomere-related genes, thereby improving myofibrillar organization and function [80].

Wang and colleagues demonstrated that loss of lamin A/C in stem- or progenitor-derived cardiomyocytes resulted in de-anchoring of cardiogenesis-related genes (e.g., *GATA4*, *MYL4*) from the nuclear lamina and increased chromatin accessibility [39]. This change triggered premature activation of genes promoting CM over endothelial cell fate, accompanied by early maturation, binucleation, cell cycle exit, and impaired contractility. Importantly, silencing Gata4 restored normal cardiac lineage specification.

Notably, the molecular mechanisms underlying cardiac defects differ between *LMNA* haploinsufficiency and point mutations that alter lamin A/C protein function. Pluripotent stem cell models harboring disease-causing *LMNA* variants, such as p.H222P and p.G609G, exhibited impaired cardiomyocyte differentiation without dissociation of cardiomyocyte-specific genes from the nuclear periphery or similar chromatin relaxation [39]. Consistent with these findings, prenatal CM models carrying the *LMNA* H222P variant displayed reduced H3K4 methylation levels and dysregulated expression of genes critical for cardiac development, including epithelial–mesenchymal transition regulators such as Mesp1 and Twist. These epigenetic and transcriptional alterations impaired CM differentiation and contributed to cardiac developmental abnormalities from early embryogenesis. Remarkably, pharmacological inhibition of LSD1—the enzyme responsible for demethylating H3K4me1—restored proper epigenetic landscape and rescued the cardiac phenotype in mouse models, highlighting LSD1 inhibition as a promising therapeutic strategy [33].

## 3. Genetic Modifiers

Broadly, a genetic modifier is a gene/genetic variant that does not independently cause a phenotype but alters the expressivity, penetrance, or severity of a primary gene/mutation, thereby modulating the resulting biological outcome. In the context of CMPs, genetic modifiers are loci distinct from the primary causal gene/mutation that influence phenotypic severity or disease susceptibility without being necessary or sufficient to cause disease; they constitute part of the individual’s genetic background and may interact with causal mutations, epigenetic mechanisms, and environmental exposures to shape clinical presentation and disease progression (Table 3) [81,82,83].

### 3.1. Genetic Modifiers in Hypertrophic Cardiomyopathy

Initial investigations relied largely on candidate-gene association approaches, focusing on functional single-nucleotide variants in pathways involved in myocardial growth and their relationship to clinical expression, particularly the extent of cardiac hypertrophy. These studies identified several putative modifier loci, notably in genes encoding components of the renin–angiotensin–aldosterone system. Among these, an insertion/deletion variant in *ACE1* gene was most frequently reported, showing associations with hypertrophic severity and/or adverse clinical outcomes [84,85,86].

Despite this shared conceptual framework, the studies differ substantially in their specific endpoints. Marian (1993) focused primarily on sudden cardiac death, reporting an overrepresentation of the DD genotype among HCM patients who experienced lethal arrhythmic events, thereby emphasizing *ACE* polymorphism as a potential risk modifier for malignant outcomes rather than for hypertrophy per se [84]. By contrast, Lechin et al. (1995) concentrated on cardiac morphology and demonstrated that patients carrying the DD genotype exhibited greater LV hypertrophy, implicating *ACE* genetic variation in the extent of myocardial remodeling [85]. Tesson et al. (1997) further demonstrated that the effect of *ACE* I/D polymorphism depends on the underlying sarcomeric mutation, indicating gene–gene interactions and highlighting the genetic heterogeneity of HCM [86].

Additional candidate modifier polymorphisms have been identified in inflammatory genes, such as *TNF-α* [90], vasoactive pathways including the endothelin system [92], and heat shock protein genes, notably *HSP70* [91], all of which have been associated with variability in clinical severity, particularly the extent of cardiac hypertrophy.

Polymorphisms in the VEGF signaling pathway have been associated with increased septal hypertrophy, higher LV outflow tract gradients, and impaired regional myocardial deformation in pediatric HCM, supporting the concept that genetic variants in angiogenesis-related loci may contribute to disease severity and may have potential utility for risk stratification and patient management [93,94].

In a more recent study, Chauhan and Sowdhamini [95] employed an integrative network approach to identify candidate modifier genes in CMPs, including hypertrophic and dilated forms. By combining human interactome data with heart-specific mouse knockout and transcriptomic evidence, the study revealed several putative modifiers, particularly *NOS3*, *SIRT1*, and *MMP2*, which may influence phenotypic variability in HCM beyond primary sarcomeric mutations. *NOS3* encodes endothelial nitric oxide synthase, a key regulator of vascular tone with well-established antihypertrophic and antifibrotic effects in the myocardium [97,98]. *SIRT1*, a NAD-dependent deacetylase, regulates multiple stress-response pathways, mitochondrial homeostasis, and cardiomyocyte survival, thereby influencing the myocardial response to pathological stress [99,100]. Notably, experimental evidence from mouse models of cardiac hypertrophy indicates that *SIRT1* activation stimulates autophagic pathways in cardiomyocytes and is associated with attenuation of hypertrophic growth, supporting a cardioprotective role for *SIRT1* in adverse remodeling [101]. *MMP-2* is a key mediator of extracellular matrix remodeling, regulating myocardial stiffness and structural adaptation. Increased *MMP-2* activity has been linked to heart failure and atrial fibrillation [102]. During cardiac remodeling, *MMP-2* is activated and secreted by cardiomyocytes, fibroblasts, endothelial cells, and inflammatory cells, contributing to dynamic alterations of the myocardial extracellular matrix [103,104].

Lindholm et al. applied a genome- and transcriptome-driven scoring system that integrated whole-genome sequencing, RNA expression, and prior hypertrophy-associated gene data to systematically prioritize candidate modifier genes [96]. Using this multi-omic framework, 165 genes achieved scores ≥ 6, with 51 scoring ≥ 7. High-ranking candidates included *CACNA1C* (score 10), *RYR2*, *TTN*, *ATP1A2*, *FHOD3*, *TJP2*, *CACNA1D*, and *DYNC1H1* (score 9), many of which have recognised roles in myocardial structure and electrical coupling.

Increasing evidence suggests that common genetic variants and their cumulative polygenic effects substantially contribute to disease expression and clinical heterogeneity in CMPs, likely modulated by environmental factors. In HCM, such common variation may explain up to 0.34 of disease heritability, with a more pronounced contribution observed in individuals without identifiable pathogenic sarcomeric variants [3,105,106,107,108,109,110,111,112]. A 2021 GWAS in over 2700 patients and 40,000 controls identified 12 genome-wide significant susceptibility loci, including variants near *FHOD3* and *TTN*. Although the effect of individual variants was modest (median per-allele OR~1.25), a polygenic risk score derived from these loci demonstrated that higher cumulative risk was associated with increased LV hypertrophy and earlier disease onset among sarcomeric variant carriers, highlighting the capacity of polygenic background to modulate monogenic HCM expressivity [105].

Tadros et al. (2025) conducted a meta-analysis of seven case–control HCM GWAS, representing the largest study of its kind with over 5900 cases and 68,000 controls [111]. They identified 62 loci associated with LV traits, including 20 novel signals. Notably, the proportion of variance explained by common variants was almost twofold higher in sarcomere-negative patients compared with sarcomere-positive ones. These findings suggest that sarcomere-negative HCM is largely polygenic and multifactorial, whereas sarcomere-positive cases may develop with fewer additional risk alleles.

Recent data support a continuum model of CMP etiopathogenesis, extending from classical monogenic disease to predominantly polygenic inheritance, with intermediate-effect variants bridging these extremes [3]. Within this framework, Meisner et al. (2025) identified relatively common, low-penetrance sarcomeric variants (population frequency ~1:350; enriched to ~1:50 in HCM) that confer modest risk in isolation but exert additive effects when co-inherited with pathogenic sarcomere mutations, thereby amplifying disease severity [113]. Functional and population-based analyses further indicated that such variants influence cardiac structure and contractility even in the absence of overt disease. Similarly, García Hernandez et al. (2025) showed that intermediate-effect variants independently contribute to HCM risk and define a graded spectrum of phenotypic expression, with more aggressive disease observed when such variants coexist with LP/P mutations [114]. Isolated intermediate-effect variants were linked to earlier disease onset and greater hypertrophy compared with genotype-negative individuals, while their co-occurrence with monogenic variants resulted in more aggressive clinical phenotypes and worse outcomes.

### 3.2. Genetic Modifiers in Dilated Cardiomyopathy

Similar to findings in HCM, polymorphisms within the renin–angiotensin system have been implicated in DCM susceptibility, with the *ACE* DD genotype showing a significant association with increased disease risk [87,88,89].

Additionally, accumulating evidence indicates that DCM is influenced by polygenic factors that modulate disease susceptibility and clinical expression [3,106,110,115,116]. Among the first common variants associated with DCM were rs1739843 (*HSPB7*), rs2234962 (*BAG3*), rs10927875 (*ZBTB17*, intronic, near *HSPB7*), rs9262636 (*HCG22*), rs3829746 (*TTN*), rs13107325 (*SLC39A8*), rs4712056 (*MLIP*), rs2291569 (*FLNC*), rs3803403 (*ALPK3*), rs2303510 (*FHOD3*), and rs150793926 (*CACNB4*, intronic) [117,118,119,120,121,122].

Subsequent studies employed more complex designs, integrating large-scale genetic and phenotypic data from biobank cohorts to further elucidate the contribution of common variants to disease risk. In this context, Pirruccello and colleagues performed a GWAS of cardiac magnetic resonance imaging traits in 36,041 UK Biobank participants; they identified 45 novel loci associated with LV structure and function, many located near genes already implicated in Mendelian cardiomyopathies [115]. A polygenic score derived from these imaging measures successfully predicted incident DCM in the general population and modulated ventricular size and function among *TTN* truncating variant carriers, highlighting the substantial contribution of common variants to DCM pathogenesis and clinical expression.

Building on these biobank-integrated analyses, recent large-scale GWAS have further refined the molecular understanding of DCM [110,123]. Jurgens et al. (2024) analyzed 9365 DCM cases and 946,368 controls, identifying 70 genome-wide significant loci, primarily implicating genes involved in CM function and contractility, with polygenic risk scores predicting disease across different ancestries and modifying risk in carriers of rare variants [123]. Similarly, Zheng et al. (2024) studied 14,256 DCM cases and 36,203 UK Biobank participants, identifying 80 loci and 62 prioritized genes, linking common variants to key biological pathways, including members of the *TGFβ* and *Wnt* signaling pathways [124]. Additionally, polygenic burden was shown to predict DCM risk and modify penetrance among carriers of monogenic variants.

## 4. Molecular Noise

Biological systems operate through biochemical reactions governed by deterministic physical laws yet exhibiting intrinsically stochastic dynamics at the molecular scale. Even genetically identical cells in homogeneous environments exhibit heterogeneous mRNA abundances, a phenomenon commonly referred to as gene expression noise. Random fluctuations in transcription, translation, chromatin dynamics and signaling arise from the discrete nature of molecular interactions, finite copy numbers of reactants and probabilistic reaction timing, generating cell-to-cell variability in gene expression and phenotype [125,126,127].

Gene expression variability arises from two conceptually distinct sources of stochasticity, notably the intrinsic noise, driven by the probabilistic dynamics of molecular reactions within gene regulatory units, and extrinsic noise, arising from fluctuations in global cellular states and regulatory factors that modulate the activity of multiple genes [125]. Gene expression noise is the stochastic variability in gene expression levels across genetically identical cells under identical environmental conditions. Four sources of protein-level variation in gene expression can be distinguished: (i) intrinsic stochastic fluctuations of molecular reaction kinetics within gene regulatory units, driven by low copy numbers and probabilistic reaction events; (ii) variability arising from heterogeneity in global cellular states across an isogenic population; (iii) spatial and temporal microenvironmental heterogeneity, including morphogen gradients; and (iv) genetic variation generated by ongoing mutational processes [126].

Early studies of stochastic gene expression in eukaryotes revealed patterns of variability that could not be explained by simple stochastic models of transcription and translation [128,129,130]. Experimental data instead support transcriptional burst models in which genes stochastically switch between discrete active and inactive states, introducing an additional regulatory layer beyond molecular reaction noise and implicating eukaryote-specific regulatory mechanisms, with chromatin dynamics as a leading candidate [131,132,133,134]. Chromatin remodeling has been widely proposed as a key driver of burst dynamics, potentially mediating stochastic transitions between transcriptionally active and inactive states [128,130,135,136], consistent with the slow, long-range dynamics of chromatin reorganization observation in vivo [137]. However, alternative mechanisms have also been implicated, including transient formation of pre-initiation complex that enable episodic rounds of transcription [129,138] and competition for transcriptional complexes that spatially concentrate RNA polymerase II activity [139,140]. In mammalian, the link between TATA box-containing promoters and increased transcriptional variability has been repeatedly confirmed [141]. Genes with low basal activity and high nucleosome occupancy near the transcriptional start site (TSS), as well as genes containing TATA boxes in their promoter regions, are consistently associated with elevated gene expression noise [142,143]. In contrast, promoters lacking CpG islands or characterized by shorter CpG-regions tend to exhibit reduced expression noise [144,145].

Mutations can directly modulate gene expression noise by altering regulatory elements and chromatin-associated processes, thereby changing the stochastic dynamics of promoter activation [130]. Both cis- and trans-acting mutations have been shown to modify noise levels without necessarily affecting mean expression, indicating that stochasticity is an evolvable trait subject to genetic control. In particular, mutations in promoter sequences and chromatin-remodeling components can increase or decrease intrinsic noise by perturbing the rates of promoter-state transitions and transcriptional activation, demonstrating that genetic variation can reshape the probabilistic architecture of gene expression rather than merely shifting expression levels [130].

Studies of synthetic genetic networks have revealed that gene expression noise is not merely a local property but propagates and transforms across regulatory architectures. In transcriptional cascades, stochastic variability in upstream genes can be transmitted to downstream targets, substantially contributing to overall expression noise, while longer cascades filter fast fluctuations but amplify temporal variability in signal propagation [146,147,148]. Feedback regulation further reshapes noise dynamics: negative feedback buffers stochastic fluctuations and stabilizes expression levels, whereas positive feedback can amplify noise and generate bistable expression states, enabling stochastic switching between phenotypic states [149,150,151,152,153].

In eucaryotes, gene expression noise is predominantly extrinsic, generating correlated fluctuations across genes and arising from heterogeneity in cellular states, upstream regulatory factors, and genomic location, yet its mechanistic basis remains incompletely understood [128,130,154,155]. At the organismal level, this intrinsic stochasticity of molecular regulation contributes to variable phenotypic outcomes in genetic disease, such that the same mutation can produce distinct clinical manifestations (variable expressivity), while a substantial fraction of mutation carriers shows no detectable pathology (incomplete penetrance). These observations challenge the traditional deterministic mapping between genotype and phenotype and instead support a fundamentally stochastic relationship between genetic variation and phenotypic outcomes [156,157]. Contemporary models of genetic interactions therefore conceptualize phenotypic traits as probabilistic distributions rather than fixed outcomes [81,158]; however, the mechanisms through which stochastic genotype–phenotype relationships emerge, their quantitative properties, and their implications for disease prediction and treatment remain poorly understood. In clinical settings, the identification of causal genetic factors is further complicated by heterogeneity in genetic backgrounds and environmental contexts, underscoring the need for frameworks that explicitly incorporate stochasticity into models of disease risk and phenotypic variability [81,156,158].

### Molecular Noise in Dilated and Hypertrophic Cardiomyopathies

CMPs illustrate a fundamentally probabilistic mapping between genotype and phenotype, in which pathogenic variants in core sarcomere and regulatory genes (Table 4), are modulated by multiple layers of molecular, cellular and genetic variability. At the molecular level, intrinsic stochasticity arising from transcriptional bursting, allele-specific expression and low-copy-number biochemical reactions generates heterogeneous expression of disease-associated genes across individual CMs [125,127,159]. Extrinsic sources of variability, including fluctuations in transcription factor abundance, chromatin states and cellular metabolic environments, further shape gene expression dynamics at the cellular and tissue levels [126].

Transcriptional bursting was demonstrated for key sarcomeric genes implicated in HCM, and was shown to generate intrinsic transcriptional noise and allelic imbalance at the single-cell level [160,164,165]. Kraft and colleagues showed that mutated and wild-type alleles of *MYH7* are transcribed independently in random bursts, resulting in dynamic allelic imbalance within individual CMs and substantial cell-to-cell contractile variability [164]. Such heterogeneity may disrupt myocardial synchronicity, promote CM disarray, and activate pro-hypertrophic and pro-fibrotic signaling pathways [165]. In heterozygous patients, this stochastic expression affects disease-causing mutations such as *MYBPC3* splice-site and truncation variants that lead to haploinsufficiency of cardiac myosin-binding protein C, and missense mutations in *TNNI3* that alter calcium sensitivity of the contractile apparatus. Independent burst-like transcription of mutant and wild-type alleles produced highly variable mutant-to-wild-type mRNA and protein ratios among individual CMs, resulting in mosaic functional heterogeneity and marked variability in calcium-dependent force generation. This intercellular variability provides a plausible mechanistic basis for focal disease onset and non-uniform myocardial remodeling frequently observed in HCM [160].

HCM is also characterized by pronounced variability in allelic expression of mutant and wild-type transcripts and proteins. In the *MYH7* gene, heterozygous mutations frequently lead to deviations from the expected 1:1 ratio of mutant and wild-type alleles at both mRNA and protein levels, with imbalance detectable at tissue and single-cell resolution [159]. Importantly, allelic imbalance is not restricted to disease states: balanced and imbalanced *MYH7* expression were observed in both HCM patients and non-HCM individuals, indicating that unequal allelic expression is an intrinsic regulatory property of this gene rather than a mutation-specific phenomenon.

Single-cell and single-nucleus transcriptomic studies have demonstrated substantial cell-to-cell heterogeneity among CMs in human CMPs, indicating that genetically similar cells can adopt distinct transcriptional states within diseased myocardium. Single-nucleus RNA sequencing of hypertrophic and dilated cardiomyopathy hearts revealed discrete CM subpopulations characterized by divergent expression programs involving sarcomeric genes such as *MYH7*, *MYBPC3* and *TTN*, as well as stress-response and remodeling pathways [161]. Similarly, single-cell transcriptomic profiling of failing human hearts uncovered marked transcriptional diversity across CMs and non-myocyte populations, reflecting heterogeneous cellular responses to pathological stress [166]. Disease-specific remodeling of gene expression programs across cardiac cell types has also been documented in DCM, further supporting the presence of divergent molecular trajectories within shared genetic contexts [162]. At the molecular level, stochastic allele-specific expression of *MYH7* has been shown to generate variable mutant-to-wild-type transcript ratios across tissues, providing direct evidence that intrinsic regulatory variability contributes to phenotypic heterogeneity in HCM [159]. Moreover, single-cell transcriptional heterogeneity is cell-type specific in human DCM: CMs largely converge toward shared disease states (notably *NPPA/NPPB* and *ANKRD1* up, *MYH6* down), whereas fibroblasts and myeloid cells diversify into multiple disease-associated subpopulations [162]. Diversification is exemplified by inflammatory myeloid programs (*CCL3/CCL4*, *IL1B*, *NLRP3*, *NFKB2*) and activated/remodeling fibroblast states (*POSTN*, *FAP*, *CTGF*, *COL1A1*, *LUM/BGN*). In contrast, endothelial and perivascular populations show broad expression shifts with limited added state complexity, indicating that heart failure remodels transcription either by state convergence (CMs) or state expansion (stromal/immune cells) [162].

Mutation-dependent expression variability in human HCM reflects cell-type-specific alterations in gene regulatory networks rather than a uniform transcriptional response to genetic variation [163]. Single-nucleus transcriptomic profiling of *MYBPC3* mutation carriers revealed extensive differential gene expression between mutation-positive and mutation-negative cardiac cells, affecting pathways involved in muscle development, contractility, calcium homeostasis, and energy metabolism. Importantly, although these biological processes were consistently perturbed, the genes driving them differed across cell types, indicating that *MYBPC3* mutations modulate regulatory networks in a context-dependent and probabilistic manner [163]. This architecture of mutation-driven transcriptional variability provides a mechanistic explanation for phenotypic heterogeneity and incomplete penetrance in human CMP, linking genetic variation to stochastic and cell-state-dependent gene expression landscapes. Building on this, snRNA-seq studies of human genetic CMPs show that pathogenic variants reshape cell-state landscapes and tissue composition, not only CM gene programs. Diseased ventricles are typically marked by CM depletion with relative expansion of endothelial and immune populations, while fibrosis reflects transcriptional state switching in fibroblasts toward extracellular-matrix remodelling rather than a simple increase in fibroblast number [161]. Within CMs, stress, metabolic and electrophysiologic programmes recur, but a substantial fraction of differential expression remains genotype- and cell-type-enriched, consistent with variant-specific reconfiguration of intercellular signalling and state occupancy. Together, these data support a model in which CMP mutations (including *MYBPC3*, *MYH7* and *TTN*) bias probabilistic transitions among pre-existing and disease-associated cell states, amplifying expression variability and producing heterogeneous trajectories at the organ level [161].

Importantly, recent data indicate that burst-like transcription, allelic imbalance, and contractile heterogeneity are detectable at early stages of disease development, preceding overt disease manifestations. Moreover, hiPSC-derived CMs from various HCM subjects consistently exhibit allelic and contractile imbalance despite controlled experimental conditions and identical underlying mutations within each line [167,168]. As protein quality control mechanisms decline with ageing, this deterioration is likely to drive an age-dependent increase in allelic imbalance and cell-to-cell contractile variability, thereby contributing to disease progression [169].

By converting stochastic fluctuations in gene expression into functional and mechanical differences, molecular noise offers a mechanistic framework for incomplete penetrance, variable expressivity, and age-dependent disease progression.

## 5. Clinical Implications, Gaps in Knowledge and Future Directions

Recognition of the contributory roles of epigenetic regulation, genetic modifiers, and molecular noise in the pathogenesis of cardiomyopathies has important clinical and translational implications. Disease expression appears to result from the cumulative effects of rare pathogenic variants, common genetic variation, epigenetic states, and environmental influences, rather than from single causative mutations alone. This framework supports more refined risk stratification strategies that integrate polygenic risk scores, modifier alleles, and potentially epigenetic markers alongside primary variants, thereby improving genetic counseling and individualized surveillance. From a clinical management perspective, elucidating modifier pathways may uncover therapeutic targets capable of modulating disease penetrance, severity, or progression independently of the primary genetic defect. Such targets could enable more personalized interventions, including tailored pharmacological strategies informed by an individual’s broader genetic and regulatory landscape. In parallel, these insights argue for expanded genomic profiling approaches that encompass both rare and common variants and, in the future, may incorporate transcriptomic and epigenomic data to better capture biological heterogeneity.

Despite these advances, significant knowledge gaps remain. The relative contributions and interactions of genetic modifiers, epigenetic mechanisms, and stochastic molecular variation are incompletely defined, and causal relationships are often difficult to distinguish from downstream or compensatory changes. Moreover, standardized approaches for integrating multi-layered genomic data into clinical decision-making are lacking, and the clinical utility of many proposed modifiers has yet to be validated.

Future research should prioritize sex-stratified, large-scale multi-omics studies integrating genomic, epigenomic, and transcriptomic data with detailed longitudinal phenotyping. Experimental validation of candidate modifiers and their interaction with primary pathogenic variants will be essential for translation into practice. Targeting true modifiers of disease severity may offer variant-agnostic therapeutic opportunities and represents a promising direction for precision medicine in cardiomyopathies.

Importantly, advances in molecular therapeutics now create unprecedented opportunities to translate mechanistic insights into precision interventions.

Adeno-associated virus (AAV)-mediated gene therapy represents a promising strategy for cardiomyopathies driven by haploinsufficiency or loss-of-function variants [170]. Cardiac-tropic AAV serotypes enable targeted myocardial gene delivery, raising the possibility of restoring deficient protein expression or modulating downstream regulatory circuits. However, challenges related to vector capacity, long-term expression, immune responses, and dose-dependent toxicity require continued refinement [171].

Genome editing technologies further expand the therapeutic landscape [172]. CRISPR–Cas-based approaches offer the potential to correct pathogenic variants directly at their genomic locus. Allele-specific editing strategies may allow selective inactivation of the mutant allele while preserving wild-type function. Nonetheless, concerns regarding off-target effects, mosaicism, delivery efficiency in post-mitotic cardiomyocytes, and long-term safety remain critical considerations [173,174].

Next-generation precision editing platforms—including base editors and prime editing—may overcome some limitations of conventional double-strand break-based genome editing [175]. Base editors enable single-nucleotide conversions without inducing double-strand DNA breaks, offering a potentially safer strategy for correcting recurrent point mutations. Prime editing further broadens the spectrum of editable variants by enabling targeted insertions, deletions, and all possible base substitutions with reduced reliance on donor templates. In cardiomyopathies characterized by recurrent missense variants, these technologies may allow highly specific correction while minimizing genomic instability. However, efficient myocardial delivery systems, durable expression control, and comprehensive assessment of off-target transcriptomic or epigenomic effects remain essential prerequisites for clinical translation.

Beyond direct gene correction, epigenome editing represents an emerging strategy to modulate disease modifiers without permanently altering DNA sequence. Such approaches may allow fine-tuning of pathogenic signaling pathways, attenuation of maladaptive remodeling, or stabilization of protective transcriptional states [172,176], thereby directly addressing the regulatory dimension of cardiomyopathy pathophysiology emphasized in this review. By moving beyond a mutation-centric model toward a systems-level understanding of penetrance and variability, future precision interventions may combine variant correction, modifier modulation, and stabilization of regulatory networks. Targeting true determinants of disease severity—rather than solely the initiating mutation—represents a compelling and biologically coherent direction for next-generation cardiovascular precision medicine.

## 6. Conclusions

Overall, current evidence supports a non-binary model of pathogenicity in dilated and hypertrophic cardiomyopathies, in which disease expression reflects a continuum of effect sizes shaped by cumulative genetic, epigenetic, and molecular stochastic influences.

## Figures and Tables

**Table 1:** List of genes with definitive, strong or moderate HCM/DCM association.

Gene–Disease Validity Classification	HCM [7]	DCM [8]
Definitive	*ACTC1*, *CSRP3*, *FHOD3*, *MYBPC3*, *MYH7*, *MYL2*, *MYL3*, *TNNC1*, *TNNI3*, *TNNT2*, *TPM1*	*BAG3*, *DES*, *FLNC*, *LMNA*, *MYH7*, *PLN*, *RBM20*, *SCN5A*, *TNNC1*, *TNNT2*, *TTN*
Strong	*ALPK3*	*DSP*
Moderate	*JPH2*, *KLHL24*, *MT-TI*, *TRIM63*	*ACTC1*, *ACTN2*, *JPH2*, *NEXN*, *TNNI3*, *TPM1*, *VCL*

**Table 2:** Epigenetic mechanisms in cardiomyopathies.

Mechanism	Genes	Key Finding	Ref.
DNA methylation	*ESR1*, *STC2*, *TGFA*, *TGFB2*, *VEGFC*, *CACNA1A*	Coordinated methylation–expression changes; immune and electrophysiological clusters	[16]
DNA methylation	*TPM1*	Promoter hypermethylation in HCM myocardium	[17]
DNA methylation	*TCAP*, *ACTA1*	Increased gene-body methylation in sarcomeric regulators	[18,19]
DNA methylation	*AUTS2*, *BRSK2*, *PRRT1*, *SLC17A7*, *GIGYF1*, *CBFA2T3*, *HIVEP3*, *ANKRD33B*, *PAX8*, *GATA4*, *PIWIL2*	Locus-specific hypomethylation linked to altered transcription	[20]
DNA methylation	*LY75*, *ERBB3*, *HOXB13*, *ADORA2A*	Differential CpG methylation in idiopathic DCM	[21]
DNA methylation	*B9D1*, *DCLK2*, *NTM*, *TBX5*, *MED13L*, *MYL2*	Cardiac- and blood-overlapping CpG biomarkers; developmental loci affected	[22]
EWAS/methylation-expression coupling	*LTBP2*, *ABHD12*, *ATP5MF*	CpG sites linked to gene expression and remodeling pathways	[23]
DNA methylation (splicing regulation)	*TTN-AS1*, *TTN*	Intronic methylation associated with exon inclusion in titin locus	[24,25,26]
DNA methylation	*SCN5A*	Altered cardiac electrical stability	[27]
DNA methylation	*TP53* (among numerous genes involved in cell death/survival, cell cycle regulation, and metabolic pathways)	LAD reorganization in LMNA-DCM associated with changes in CpG methylation and extensive dysregulation of coding and noncoding genes	[28]
Histone modification	*RAS–MAPK* pathway	Coexistence of activating and repressive histone modifications	[29,30]
Histone modification/chromatin remodeling	*MYH6*, *MYH7*	BRG1-mediated α/β-MHC isoform switch; fetal reprogramming	[31,32]
Histone demethylation imbalance	*MESP1*, *TWIST*	Reduced H3K4 methylation impairs cardiac differentiation	[33]
Histone deacetylation	*CACNA1H*, *CACNA2D2*, *ACTA1*, *TNNT3*, *TNNI1*, *TNNI2*	HDAC1/2 loss induces calcium-handling and skeletal gene reprogramming	[34]
Histone methylation	*DMD*	DOT1L-dependent regulation of dystrophin stability	[35,36]
Chromatin architectural remodeling	*HMGN3*, *HMGA1*, *HMGA2*, *ZMYND8*, *DPF3*, *H2BC7*, *H3C12*, *H4C16*, *IL1A*, *IL1B*, *CXCL8*, *VEGFA*, *BNIP3*, *CA9*, *SLC2A1*, *TP53I3*, *GLIPR1*, *FAS*, *BBC3*, *BTG2*, *DES*, *ACTA2*, *LMOD1*, *TMOD1*, *OBSCN*, *MYOM1*, *PKP2*, *KCNJ11*, *KCNJ14*, *CACNA2D2*	HMGN3 loss alters H3K27ac distribution and exerts bidirectional transcriptional effects, such as upregulation of chromatin-remodelling factors, activation of inflammatory, hypoxic, and apoptotic pathways, while suppressing survival and cardiac genes.	[37]
Chromatin accessibility	*SP1*, *EGR1*	Fetal-like TF motif enrichment and increased chromatin accessibility	[38]
Lamina–gene interaction loss	*GATA4*, *MYL4*	De-anchoring from nuclear lamina increases chromatin accessibility	[39]
Open chromatin activation	*PDGFRB*	Aberrant PDGF signaling in LMNA haploinsufficiency	[40]
LAD redistribution/chromatin remodeling	*SCN5A*, *KCNA5*, *CACNA1A/C/D*, *HCN4*, *SCN3B*, *SCN4B*	Ion channel dysregulation secondary to lamin-associated chromatin reorganization	[27,39,40,41,42]

**Table 3:** Genetic modifiers in cardiomyopathies.

Mechanism	Genes	Key Finding	Ref.
Genetic modifier	*ACE*	DD genotype associated with hypertrophy severity and adverse outcomes, and DCM susceptibility	[84,85,86] [87,88,89]
Genetic modifiers	*TNF*, *HSP70*	Inflammatory and stress-response polymorphisms modulate disease severity	[90,91,92]
Genetic modifiers	*VEGF*	Angiogenesis-related polymorphisms associated with more severe phenotype in pediatric HCM	[93,94]
Genetic modifiers	*NOS3*, *SIRT1*, *MMP2*	Candidate modifiers influencing hypertrophy and ECM remodeling	[95]
Genetic modifiers	*CACNA1C*, *RYR2*, *TTN*, *ATP1A2*, *FHOD3*, *TJP2*, *CACNA1D*, *DYNC1H1*	High-scoring candidate modifier genes by multi-omic analysis	[96]

**Table 4:** Examples of molecular noise sources and mechanisms in cardiomyopathies.

Noise Level	Mechanism of Noise	Cardiomyopathy Type	Key Genes Involved	Evidence of Stochasticity	References
Intrinsic transcriptional noise	Transcriptional bursting	HCM	*MYBPC3*, *TNNI3*, *MYH7*	Single-cell variability and burst-like transcription of sarcomeric genes	[160]
Allelic expression imbalance	Stochastic allele-specific expression	HCM	*MYH7*	Variable mutant vs. wild-type allele expression across cells and tissues	[159]
Cell-to-cell transcriptional variability	Single-cell transcriptomic heterogeneity	DCM	*MYH7*, *TTN*, *LMNA*	Distinct cardiomyocyte transcriptional states and heterogeneous gene expression profiles revealed by single-nucleus RNA-seq in cardiomyopathy hearts	[161]
Single-cell transcriptional heterogeneity	Disease-specific cell states	DCM/	*NPPA*, *NPPB*, *ANKRD1MYH6*	Divergent cardiomyocyte transcriptional programs in diseased hearts	[162]
Mutation-dependent expression variability	Cross-cell-type variability	HCM	*MYBPC3*	Variable expression patterns across cardiomyocytes and species	[163]
Cell-state diversification	Altered cellular composition	DCM	*MYH7*, *TTN*	Heterogenous cardiac cell populations	[161]

## Data Availability

No new data were created or analyzed in this study. Data sharing is not applicable to this article.

## Abbreviations

The following abbreviations are used in this manuscript:

*ABHD12*	abhydrolase domain containing 12
*ACE1*	angiotensin 1 converting enzyme
*ACTA1*	actin alpha 1
*ACTA2*	actin alpha 2
*ADORA2A*	adenosine receptor A2A
*ANKRD33B*	ankyrin repeat domain 33B
ANP	atrial natriuretic polypeptide
ARVC	arrhythmogenic right ventricular cardiomyopathy
AS	aortic stenosis
*ATP5MF*	ATP synthase membrane subunit f
*ATP11A*	ATPase phospholipid transporting 11A
*AUTS2*	autism susceptibility candidate 2
*B9D1*	B9 domain-containing protein 1
*BBC3*	Bcl-2-binding component 3
*BNIP3*	BCL2/adenovirus E1B 19-kDa-interacting protein 3
*BNP*	brain-type natriuretic peptide
*BRG1*	brahma-related gene 1
*BRSK2*	BR serine/threonine kinase 2
*BTG2*	B-cell translocation gene 2
*CA9*	carbonic anhydrase 9
*CACNA1A*	calcium voltage-gated channel subunit alpha1 A
*CACNA1H*	calcium voltage-gated channel subunit alpha1 H
*CACNA2D2*	calcium voltage-gated channel auxiliary subunit alpha2delta 2
*CBFA2T3*	CBFA2/RUNX1 partner transcriptional co-repressor 3
CM	cardiomyocyte
CMP	cardiomyopathy
*CXCL8*	C-X-C motif chemokine ligand 8
*DCLK2*	doublecortin like kinase 2
DCM	dilated cardiomyopathy
DES	desmin
Dmd	dystrophin
DMR	differentially methylated region
DNAme	DNA methylation
*DPF3*	double PHD fingers 3
*EGR1*	early growth response 1
*ERBB3*	tyrosine kinase-type cell surface receptor HER3
*ERC2*	ELKS/RAB6-Interacting/CAST Family Member 2
*ESR1*	estrogen receptor 1
EWAS	epigenome-wide association study
FAO	fatty acid oxidation
*GATA4*	GATA-binding protein 4
*GIGYF1*	GRB10-interacting GYF protein 1
*GLIPR1*	glioma pathogenesis-related protein 1
GO	gene ontology
*H2BC7*	H2B clustered histone 7
*H3C12*	H3 clustered histone 12
*H4C16*	H4 clustered histone 16
HATs	histone acetyltransferases
HCM	hypertrophic cardiomyopathy
*HCN4*	hyperpolarization-activated cyclic nucleotide-gated channel 4
HDACs	histone deacetylases
HDMs	histone demethylases
hiPSC-CMs	induced pluripotent stem cell-derived cardiomyocytes
*HIVEP3*	human immunodeficiency virus type 1 enhancer-binding protein 3
*HMGA1/2*	high mobility group at-hook 1
*HMGN*	high mobility group nucleosome-binding
HMTs	histone methyltransferases
HOCM	hypertrophic obstructive cardiomyopathy
*HOXB13*	homeobox B13
*KCNA5*	potassium voltage-gated channel subfamily a member 5
*KCNJ11*	potassium inwardly rectifying channel subfamily j member 11
*KCNJ14*	potassium inwardly rectifying channel subfamily J member 14
*IL1A*	interleukin 1 alpha
*IL1B*	interleukin 1 beta gene
KEGG	Kyoto encyclopedia of genes and genomes
LADs	lamin-associated domains
*LMNA*	laminin
*LMOD1*	leiomodin 1
*LRRC10*	leucine-rich repeat containing protein 10
*LRRC14B*	leucine-rich repeat-containing protein 14B
*LTBP2*	latent transforming growth factor beta binding protein 2
LV	left ventricular
LVAD	left ventricular assist device
*LY75*	lymphocyte antigen 75
*MAPK*	mitogen-activated protein kinase
*MED13L*	mediator complex subunit 13l
*MLC1F*	myosin light chain 1
*MMP2*	matrix metalloproteinase-2
*MYH6*	myosin heavy chain 6
*MYL2*	myosin light chain 2
*MYOM1*	myomesin 1
NDLVC	non-dilated left ventricular cardiomyopathy
*NEB*	nebulin
*NOS3*	nitric oxide synthase 3
*NTM*	neurotrimin
*OBSCN*	obscurin
OXPHOS	oxidative phosphorylation
*PAX8*	paired box gene 8
*PIWIL2*	piwi-like RNA-mediated gene silencing 2
*PKP2*	plakophilin-2
*PRRT1*	proline-rich transmembrane protein 1
*PSORS1C3*	psoriasis susceptibility 1 candidate 3
*RBM19*	RNA binding motif protein 19
RCM	restrictive cardiomyopathy
*SCN3B*	sodium voltage-gated channel beta subunit 3
*SCN4B*	sodium voltage-gated channel beta subunit 4
*SCN5A*	sodium voltage-gated channel alpha subunit 5
*SIRT1*	sirtuin 1
*SLC2A1*	solute carrier family 2
*SLC12A7*	solute carrier family 12 member 7
*SLC17A7*	solute carrier family 17 member 7
SMR	summary-based Mendelian randomization
*SP1*	plicamycin
*STC2*	stanniocalcin 2
*TBX3*	T-box transcription factor 3
*TBX5*	T-box transcription factor 5
*TBX5-AS1*	T-box transcription factor 5 antisense RNA 1
*TCAP*	titin-cap
*TGFA*	transforming growth factor alpha
*TGFB2*	transforming growth factor beta 2
*TMOD1*	tropomodulin 1
*TNNI1*	troponin I1
*TNNI2*	troponin I2
*TNNT3*	troponin T3
*TP53*	tumor protein p53
*TPM1*	tropomyosin 1
*TPM3*	tropomyosin 3
*TSPAN9*	tetraspanin 9
TSS	transcriptional start site
*TTN*	titin
*TTN-AS1*	RNA titin antisense 1
*VEGFA*	vascular endothelial growth factor A
*VEGFC*	vascular endothelial growth factor C
WT	wild-type
*ZMYND8*	zinc finger mynd-type containing 8
